# Epithelioid Hemangioma of the Glans Penis in a Preschool-Aged Child: A Case Report

**DOI:** 10.3390/children13101418

**Published:** 2026-10-16

**Authors:** Carlos Andrés Yánez Ruiz, Mario Daniel Tapia Tapia, Andrés Calva López, Vanessa Talavera Cobo, Francisco Javier Ancizu Marckert, Shirley Amarilis Lorenzo Meza, Daniel Sánchez Zalabardo, José Enrique Robles García

**Affiliations:** 1 Department of Urology, Clínica Universidad de Navarra, 31008 Pamplona, Spain; cyanezruiz@unav.es (C.A.Y.R.); mdtapia@unav.es (M.D.T.T.); acalva@unav.es (A.C.L.); vtalaveracobo@yahoo.com (V.T.C.); fancizu@unav.es (F.J.A.M.); dsanchezz@unav.es (D.S.Z.); 2 Department of Pathology, Clínica Universidad de Navarra, 31008 Pamplona, Spain; slorenzom@unav.es

**Keywords:** epithelioid hemangioma, glans penis, pediatrics, benign vascular tumor, immunohistochemistry

## Abstract

**Highlights:**

**What are the main findings?**

• Epithelioid hemangioma of the glans penis is a rare benign vascular tumor in childhood with nonspecific clinical features.
• Diagnosis was primarily based on histopathology, with immunohistochemistry supporting endothelial differentiation.

**What are the implications of the main findings?**

• Clinically indeterminate glanular lesions in children warrant careful diagnostic evaluation, including histopathological assessment.
• For small, superficial, and well-demarcated lesions, complete local excision may offer both diagnostic confirmation and local treatment while preserving the urethral structures.

**Abstract:**

Background: Epithelioid hemangioma is an uncommon benign vascular tumor, and involvement of the glans penis is exceptional in childhood. Case Presentation: We report the case of a 4-year-old boy who presented with a longstanding, painless, pedunculated pink nodule on the dorsal glans adjacent to the urethral meatus. Previous topical treatment for presumed balanitis relieved pruritus but did not modify the lesion. Complete local excision was performed under general anesthesia with local infiltration, with preservation of the urethral structures. Histopathological examination showed a well-circumscribed polypoid mucosal lesion with epithelioid endothelial proliferation, small vascular channels, and mild stromal inflammation, without cytological atypia. Immunohistochemistry showed strong FLI-1 and CD34 positivity, supporting endothelial differentiation; CD31 was not performed. The morphological features strongly favored benign epithelioid vascular proliferation and were not consistent with epithelioid hemangioendothelioma or epithelioid angiosarcoma. The postoperative course was uneventful, and follow-up at 12 months showed no recurrence. Conclusions: This case illustrates the need to consider rare vascular tumors in persistent pediatric penile lesions. A treatment-refractory glanular lesion in a child should prompt early histopathological assessment rather than prolonged empirical therapy.

## 1. Introduction

Epithelioid hemangioma is a rare benign vascular tumour characterised by vascular spaces lined by plump epithelioid endothelial cells, usually accompanied by a variable inflammatory infiltrate. Although it has a recognised predilection for the head and neck region, penile involvement is unusual, and cases arising on the glans in children are especially scarce [1,2]. Reported penile lesions have been described as small nodules, papules or vascular-appearing masses, with symptoms ranging from local tenderness or bleeding to entirely asymptomatic presentations [3].

The clinical differential diagnosis of a persistent glanular lesion in childhood is broad and includes balanitis, pyogenic granuloma, angiokeratoma, lymphatic or venous malformations and epithelial malignancy, as well as malignant vascular tumours such as epithelioid hemangioendothelioma and epithelioid angiosarcoma [4,5]. Histopathology remains the basis for diagnosis, and immunohistochemistry is particularly useful in morphologically challenging cases, because endothelial markers help confirm vascular lineage and support exclusion of mimics with more aggressive behavior [6,7,8]. This report describes a preschool-aged boy with a glanular epithelioid hemangioma that was successfully managed by complete excisional biopsy with primary closure and preservation of the urethral structures. The distinguishing features of this case are the dorsal glanular location, preschool age, acquired (non-congenital) presentation, solitary nature, and tissue-sparing surgical management with no recurrence at 12 months. We also summarize the previously reported pediatric cases of penile epithelioid hemangioma to contextualize the contribution of this report (Table 1).

## 2. Case Presentation

### 2.1. Clinical Presentation

A 4-year-old Caucasian boy from Navarra, Spain, with no relevant systemic disease, presented with a persistent glanular lesion. Pregnancy and delivery were uncomplicated and at term, with a normal neonatal genital examination. The lesion was first noticed at approximately 3.5 years of age (6 months before presentation) and was therefore considered acquired, though exact onset cannot be confirmed retrospectively. He had no history of circumcision, genital surgery, recurrent balanitis, trauma, infection, or urinary symptoms, and no family history of vascular lesions. Prior topical treatment for presumed balanitis resolved pruritus but not the nodule.

Physical examination showed a redundant prepuce with balanopreputial adhesions, allowing partial exposure of the glans. On the dorsal aspect of the glans, adjacent to the urethral meatus, there was a solitary pedunculated pink lesion measuring approximately 3 mm, with a smooth surface and no tenderness on palpation. Clinical appearance of the lesion is shown in Figure 1.

No active bleeding, ulceration, or surrounding inflammatory changes were observed. Given the diagnostic uncertainty associated with a persistent, non-resolving glanular lesion in a child and the lesion’s small size (~3 mm), superficial, exophytic, and well-demarcated nature, surgical excision was performed without preoperative imaging. This approach allowed simultaneous histopathological diagnosis and definitive treatment while avoiding the potential morbidity of a separate biopsy procedure. We acknowledge that ultrasound with Doppler may be useful in larger, deeper, or equivocal lesions to characterize vascularity and assist surgical planning.

### 2.2. Histopathological and Immunohistochemical Findings

The lesion was submitted for histopathological examination after excision. Microscopy showed a polypoid mucosal lesion covered by non-keratinising squamous epithelium, without epithelial maturation abnormalities or viral cytopathic changes. The underlying stroma contained a well-demarcated vascular proliferation composed of small-calibre vascular channels and epithelioid endothelial cells with abundant eosinophilic cytoplasm and round nuclei. Mild stromal fibrosis and a sparse lymphocytic inflammatory infiltrate were present. There was no cytological atypia, atypical mitotic activity, necrosis, thrombosis or infiltrative growth pattern. Surgical margins were histologically free of lesion. Histopathological findings are shown in Figure 2.

Immunohistochemistry demonstrated strong nuclear positivity for FLI-1 and diffuse CD34 staining in the lesional endothelial cells, supporting endothelial differentiation. CD31 was not performed. Importantly, FLI-1 and CD34 are pan-endothelial markers that confirm vascular lineage but are not specific for epithelioid hemangioma and can be positive in epithelioid hemangioendothelioma and epithelioid angiosarcoma. The diagnosis was based mainly on morphological characteristics (well defined lobular growth of small-sized vessels lined by epitheliod endothelial cells without atypia, necrosis, thrombosis or invasive growth) and on immunohistochemistry as a supplementary mean to confirm endothelial origin. In correlation with morphology, these findings strongly favored a benign epithelioid vascular proliferation consistent with epithelioid hemangioma. Immunohistochemical findings are shown in Figure 3.

### 2.3. Treatment

Complete local excision was performed under general anesthesia with local infiltration of lidocaine 1% with epinephrine for haemostasis and postoperative analgesia. The lesion was resected with a 1–2 mm macroscopic margin of normal-appearing tissue to ensure complete resection while preserving urethral meatus and adjacent urethral structures. The procedure did not include preputioplasty or circumcision. The excised tissue was submitted in full for pathological assessment.

### 2.4. Outcome and Follow-Up

The postoperative course was uncomplicated, with adequate wound healing and no reported urinary difficulty. Clinical follow-up at 6 months showed no local recurrence, bleeding, meatal stenosis or cosmetic concern. At the 12-month visit, the glans demonstrated excellent healing with a symmetrical shape, without visible scarring or distortion, and a normally located, unobstructed urethral meatus. Neither of the follow-up appointments included a standardized postoperative clinical photograph; the postoperative evaluation was recorded descriptively in the clinical documentation. Continued observation was recommended because recurrence has been described after incomplete excision in pediatric penile epithelioid hemangioma.

## 3. Discussion

Epithelioid hemangioma of the penis is a rare benign vascular tumor, and it is extremely rare on the glans in children of preschool age. The largest clinicopathological series of penile lesions to date has been reviewed by Fetsch et al. The patients were adults and ranged in age from 23 to 75 years. Most lesions were solitary and dorsally located [6]. Like the others, this case is solitary and dorsal in location but is remarkable for occurring in a 4-year-old child, an age group in which this entity has been reported only in isolated case reports [7,9]. Thus, this case contributes to the scarce literature on pediatric glanular epithelioid hemangioma and emphasizes the special diagnostic and therapeutic issues in this population. Table 1 summarizes the previously reported pediatric cases and contextualizes the contribution of the present report.

A diagnostic challenge is given by the clinical presentation of a persistent, painless, pedunculated nodule on the glans in a child. The differential diagnosis is broad and includes benign vascular lesions (e.g., pyogenic granuloma, angiokeratoma), non-neoplastic inflammatory conditions (e.g., balanitis, lichen sclerosus), and most importantly malignant vascular tumors (e.g., epithelioid hemangioendothelioma, epithelioid angiosarcoma) [4,5]. In this case, the lesion was clinically considered a pyogenic granuloma due to its pedunculated and erythematous appearance, but the histopathological examination did not support this diagnosis, showing a well-circumscribed lobular vascular proliferation without the typical surface ulceration and granulation tissue that is characteristic of pyogenic granuloma [10]. Similarly, the absence of superficial ectatic vessels and epidermal hyperkeratosis argued against angiokeratoma [11]. The lack of cytological atypia, atypical mitosis, necrosis, thrombosis and infiltrative growth pattern, features typically present in epithelioid hemangioendothelioma and epithelioid angiosarcoma, argued against these entities, both of which have aggressive histology and a propensity for metastasis [4,6]. The overlying squamous epithelium showed no dysplasia, further arguing against squamous cell carcinoma [10]. Immunohistochemistry was essential to confirm endothelial lineage. Intense nuclear reactivity for FLI-1 and diffuse membranous/cytoplasmic staining for CD34 confirmed endothelial differentiation, in accordance with the previously reported immunophenotypes [6,7]; however, as these markers may also be expressed by malignant epithelioid vascular tumors, the benign diagnosis relied mainly on morphology.

Management of glanular epithelioid hemangioma in children should balance the need for definitive diagnosis and preservation of penile function and esthetics. Observation or topical therapy was not selected because the lesion was persistent, treatment refractory and of diagnostic uncertainty. Non-surgical options such as sclerotherapy or laser therapy have been successfully applied in the treatment of penile vascular anomalies, particularly venous malformations and small lesions [12,13,14]; however, these approaches do not allow tissue sampling for histopathological examination, and they are associated with the risk of incomplete treatment or recurrence. Destructive techniques such as cryotherapy, electrocautery and laser ablation were considered but dismissed due to inability to obtain tissue for histopathological evaluation, potential for incomplete treatment with resultant recurrence requiring urethral reconstruction [9] and the risk of hypopigmentation, scarring or meatal distortion. The excisional biopsy with primary closure was done allowing precise margin control and a great cosmetic result. In the setting of a persistent glanular lesion in a child, where the differential diagnosis includes rare but aggressive malignancies, excisional biopsy provides the dual benefit of a definitive diagnosis and complete local treatment [8,15]. The observation of local recurrence after incomplete excision of penile epithelioid hemangioma reported by Kohli et al. in a case that needed more extensive surgery with urethral reconstruction following recurrence in an adolescent also supports this [9]. Our favorable outcome, with no recurrence at 12 months, supports complete excision as an appropriate approach for small, superficial and well-demarcated lesions such as the one described here.

This case presents several important considerations. In the first instance, the initial treatment for presumed balanitis resulted in symptomatic relief of pruritus, but the lesion did not change. This case emphasizes the importance of re-evaluating the diagnosis when a lesion does not resolve with appropriate conservative therapy. Second, the decision to proceed directly to excision without preoperative imaging was based on the small size, superficial location and accessibility of the lesion and the need for tissue diagnosis. Imaging such as ultrasound with Doppler may be useful in characterizing vascular flow or assessing depth of invasion in larger or equivocal lesions, but was not deemed necessary in this case. Third, the absence of recurrence at 12 months is reassuring but longer surveillance is appropriate, especially as recurrence has been described after incomplete resection in pediatric patients [9]. Although reported outcomes following complete excision are favorable, continued follow-up is therefore recommended.

The management of complex or multifocal penile epithelioid hemangioma may necessitate a multidisciplinary approach and alternative treatment modalities. A case of multifocal penile epithelioid hemangioma treated successfully with doxorubicin was described by Fawzi et al. and highlighted the possible role of systemic therapy when surgery is not feasible or curative [16]. However, for solitary, well-circumscribed lesions such as the one described here, complete surgical excision represents a reasonable diagnostic and therapeutic approach.

This report has several limitations. As a single case report, it cannot establish a treatment algorithm or define recurrence risk. The follow-up of 12 months is reassuring but short and longer surveillance would be required to confirm cure. Moreover, this entity is rare in the pediatric population, hence the limitation in comparison with larger cohorts. Nevertheless, this report adds to the limited literature regarding pediatric penile epithelioid hemangioma and illustrates a successful diagnostic and therapeutic approach that may be applied to similar clinical situations.

## 4. Conclusions

Epithelioid hemangioma of the glans penis, although very rare in children, should be kept in the differential diagnosis of persistent uncharacterized glanular lesions refractory to initial conservative management. The histopathology remains the basis for distinguishing it from malignant epithelioid vascular tumors, and immunohistochemistry can support confirmation of endothelial lineage. In this case, complete local excision with preservation of urethral structures was diagnostic and curative, with no recurrence at 12-month follow-up. This approach may represent an appropriate strategy for similarly small, superficial, well-demarcated lesions, although the evidence base derives from single cases and small series. Continued clinical surveillance is advised to detect recurrence, especially after incomplete resection.

## Figures and Tables

**Figure 1:**
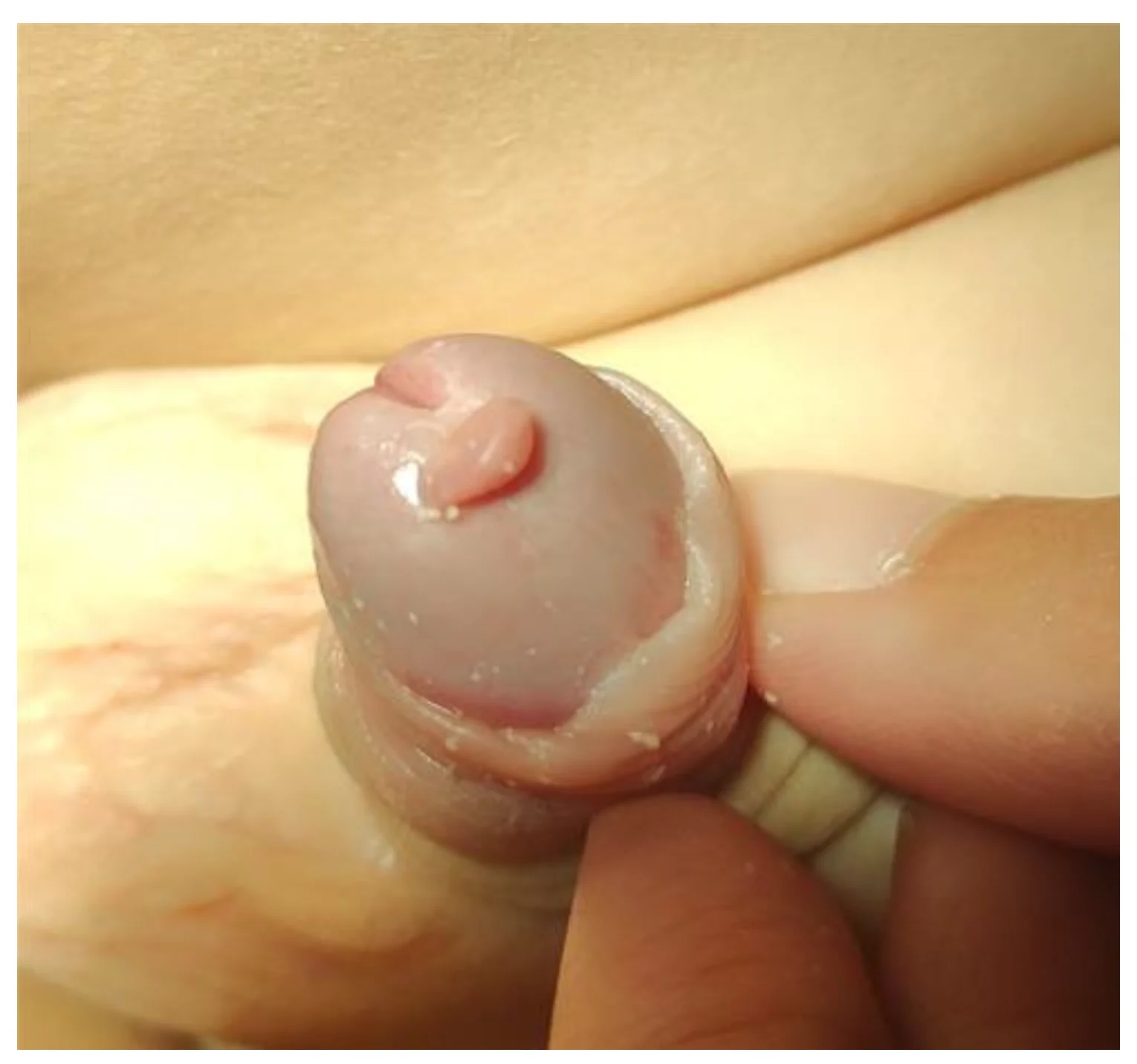
Clinical appearance of the lesion. A pedunculated pink nodule is visible on the dorsal aspect of the glans, adjacent to the urethral meatus, which is visible inferiorly, confirming the dorsal location of the lesion; the ventral surface is not visualized.

**Figure 2:**
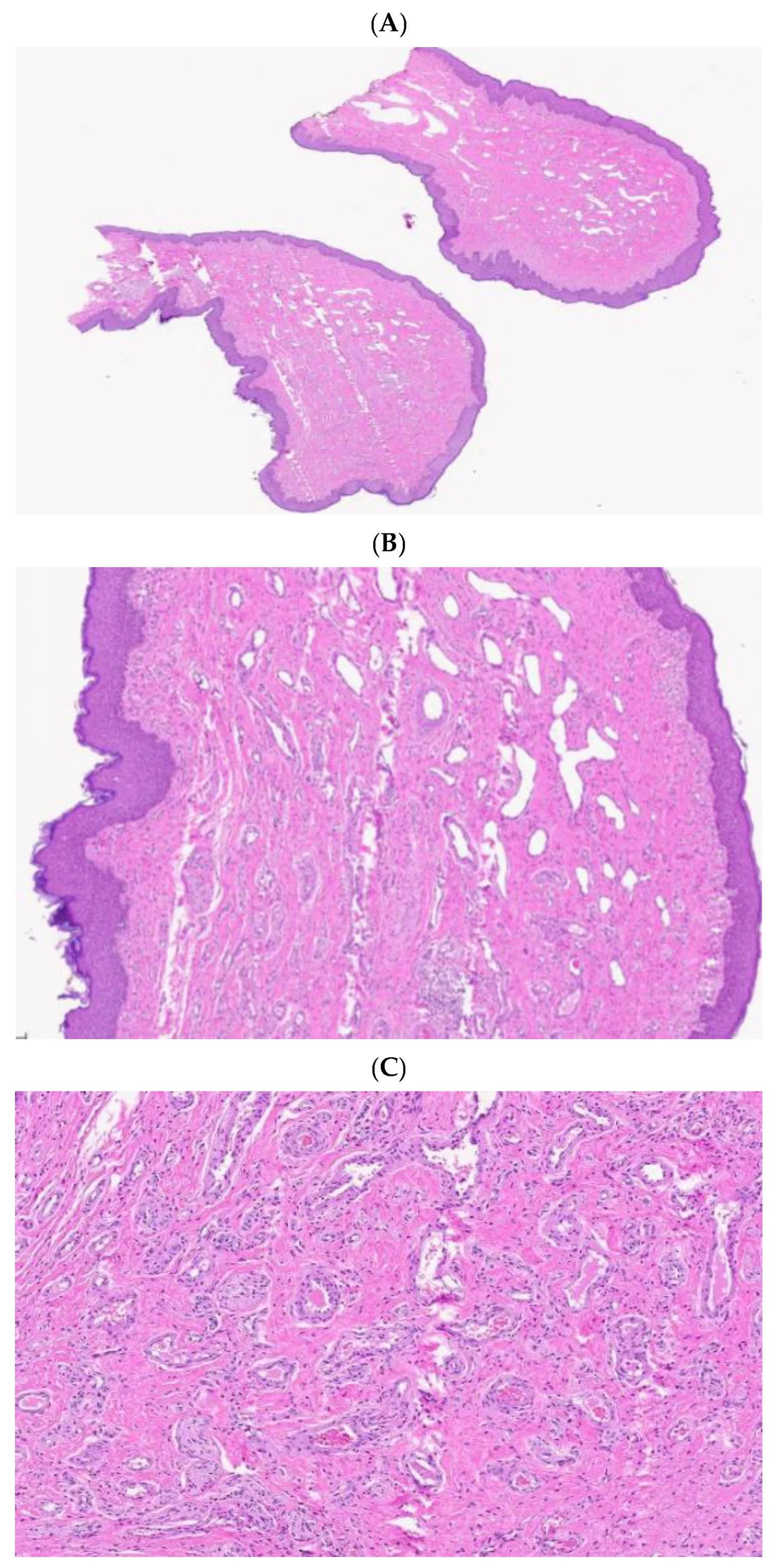
Histopathological findings with haematoxylin and eosin (H&E) staining. (**A**) Low-power view of a well-circumscribed polypoid lesion. (**B**) Intermediate-power view showing lobulated vascular architecture with small-calibre vessels. (**C**) High-power view showing epithelioid endothelial cells with round nuclei and no cytological atypia.

**Figure 3:**
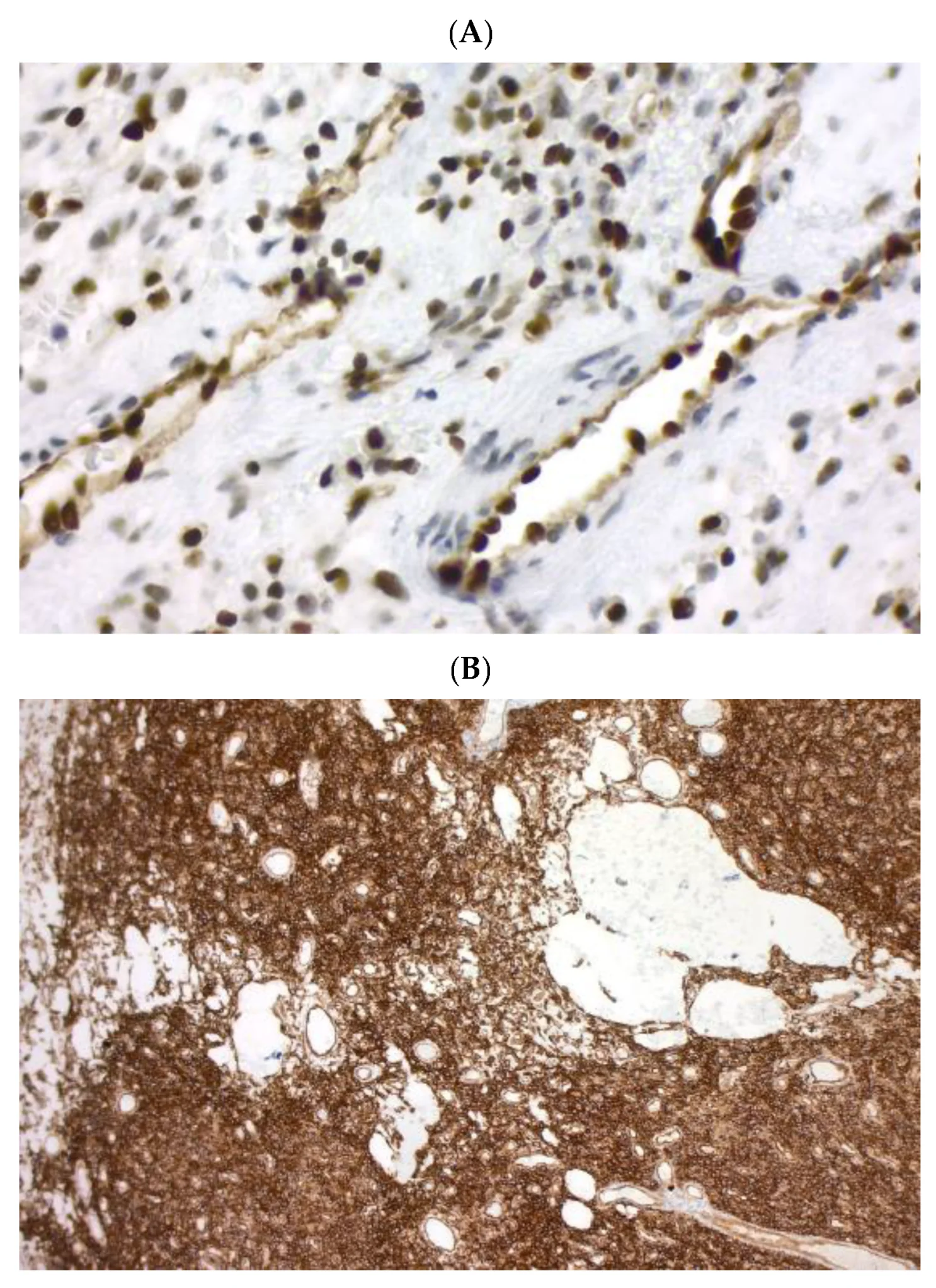
Immunohistochemical findings. (**A**) Strong nuclear positivity for FLI-1 in the epithelioid endothelial cells. (**B**) Diffuse membranous/cytoplasmic positivity for CD34 in the epithelioid endothelial cells, supporting the diagnosis of epithelioid hemangioma.

**Table 1:** Reported pediatric cases of penile epithelioid hemangioma.

Author (Year)	Age	Location	Solitary/ Multifocal	Treatment	Recurrence/ Follow-Up	Key Pathological Findings
Murshed & Farghaly (2020) [7]	4 years	Penis	Multifocal	Not specified	Not specified	Bland endothelial cells, limited inflammation
Kohli et al. (2022) [9]	Adolescent	Glans penis	Solitary	Initial resection, then more extensive surgery with urethral reconstruction for recurrence	Recurrence after initial resection	Epithelioid hemangioma
Present case (2026)	4 years	Dorsal glans	Solitary	Complete local excision under general anaesthesia with primary closure; no circumcision	No recurrence at 12 months	Well-circumscribed lobular proliferation; epithelioid endothelial cells without atypia; FLI-1+/CD34+; CD31 not performed

## Data Availability

Data supporting this case report are presented within the article. Clinical records are not publicly available due to patient confidentiality but may be obtained from the corresponding author upon reasonable request.

## Abbreviations

The following abbreviations are used in this manuscript:

H&E	Haematoxylin and Eosin
FLI-1	Friend Leukemia Integration 1
CD34	Cluster of Differentiation 34
